# Functional Traits and Phylogenetic Effects Drive Germination of Lemur‐Passed Seeds

**DOI:** 10.1002/ece3.70881

**Published:** 2025-02-04

**Authors:** Camille M. M. DeSisto, Zico Zandry, Telesy Feno, Borna Zareiesafandabadi, Jean Randrianasy, Jean Tiamanana, Dominique Randrianasolo, Manadina Rasolofo, George Raveloson, Franclin Zerimanana, Onja Razafindratsima, James P. Herrera, John R. Poulsen

**Affiliations:** ^1^ Nicholas School of the Environment Duke University Durham North Carolina USA; ^2^ Centre Universitaire Régional de la SAVA Antalaha Madagascar; ^3^ University of Antsiranana Antsiranana Madagascar; ^4^ University of North Carolina Chapel Hill Chapel Hill North Carolina USA; ^5^ Marojejy Guide Association Andapa Madagascar; ^6^ Ambodivoara Vondron'Olona Ifotony Andapa Madagascar; ^7^ University of California Berkeley Berkeley California USA; ^8^ Duke Lemur Center SAVA Conservation Durham North Carolina USA; ^9^ The Nature Conservancy Boulder CO USA

**Keywords:** functional traits, germination, gut passage, phylogeny, seed dispersal, tropical forests

## Abstract

Frugivore‐mediated seed dispersal drives ecological functioning across tropical forests. The biological mechanisms affecting seed dispersal outcomes, as well as the role of specific functional traits in plants and their dispersers, is still not well understood. To address this gap, we conducted germination experiments in eight species of captive and two species of wild lemurs, which disperse different plant species. We (1) quantified the effects of pulp removal, seed priming, and feces effects (nutrient/microbial fertilization) through gut passage as mechanisms, (2) determined the effect of frugivore species on germination, and (3) assessed how individual plant and animal traits affected two seed germination outcomes: success rates and time‐to‐germination. Accounting for phylogenetic non‐independence of plants and estimating phylogenetic signal, we evaluated the effects of lemur gut passage and functional traits in a Bayesian framework. Seed priming during gut passage was the primary mechanism through which lemurs improved germination rates and decreased time‐to‐germination. Gut passage influenced the effect of seed length on germination probability but not time‐to germination. Germination outcomes varied by disperser species and seed size. Furthermore, seeds passed by male lemurs were 40% more likely to germinate than those passed by female lemurs. Germination probability was more similar for closely related plant species compared to those that were more distantly related, while the plant phylogenetic effects on time‐to‐germination were weaker. Moreover, germination depended on experimental setting; for example, lemur gut passage decreased time‐to‐germination in captive, but not wild settings. Our results highlight the complexity of biological mechanisms determining seed dispersal outcomes; ecological and evolutionary factors were important drivers of germination. Considering a diversity of potential effects is critical for advancing a mechanistic understanding of species interactions and their outcomes.

## Graphical abstract

Seed dispersal by frugivores can alter plant germination, driving ecological functioning across tropical forests. In an experimental framework, we quantified the biological mechanisms through which gut passage by lemurs affects germination success and time‐to‐germination. Functional traits, phylogenetic effects, and environmental context determined germination, highlighting the importance of both ecological and evolutionary processes in seed dispersal outcomes.

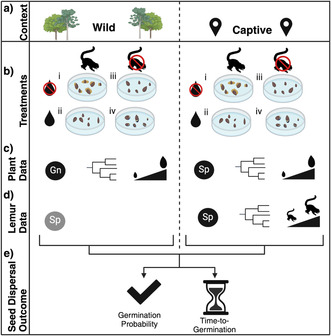

## 1 Introduction

Seed dispersal drives evolutionary, community, and ecosystem processes in tropical forests (Guimarães, Jordano, and Thompson 2011; Schleuning et al. 2014; Rogers et al. 2021). The effects of seed dispersal on plant population persistence depend on both the quantity—number of seeds dispersed—and quality—functional outcomes for seed survival and growth—of the interaction (Schupp, Jordano, and G'omez 2010, 2017). Frugivores can influence seed dispersal quality through processes that occur during gut passage, such as scarification, and by defecating intact seeds in favorable microsites, potentially improving seed germination. Germination success rates and time‐to‐germination influence plant survival, phenotypic trait expression, ecological niches, and geographic ranges (Donohue et al. 2010). Variation in the effectiveness of seed dispersal across communities predicts the ability of plants to track climate change by dispersing to new environments (Fricke et al. 2022); understanding the drivers of germination is, therefore, especially important in a time of rapid environmental change.

The effect of frugivore seed dispersal on germination is highly variable (Traveset and Verdú 2002; Rogers et al. 2021). At a global scale, frugivores significantly improve germination success rates (Traveset and Verdú 2002). Recent meta‐analyses determined that primate seed dispersal tends to increase germination success rates and decrease time‐to‐germination compared to other taxa (Rogers et al. 2021; Fuzessy, Janson, and Silveira 2018). Gut passage by frugivores can improve germination success rates and decrease time‐to‐germination through three key mechanisms: (1) pulp removal (causing deinhibition to stimulate germination), (2) seed priming (mechanical scarification and chemical enhancement of seed coat permeability to key resources), and (3) feces effects (deposition of seeds in nutrient‐dense feces along with microbial communities) (Traveset 1998; J. H. Connell 1971; Janzen 1970; Lehouck et al. 2012; Fricke et al. 2019). Understanding the mechanisms that affect seed germination is critical for predicting the effects of seed dispersal on plant performance.

Trait variation of both plants and seed dispersers also plays an important role in germination (Laughlin et al. 2020). Seed size, for example, is a well‐known driver of germination success (Schupp et al. 2019). Globally, intraspecific trait variation accounts for approximately 25% of morphological and physiological plant trait variability within communities and 32% of the trait variation among communities (Siefert et al. 2015). Despite the importance of a suite of traits for germination, studies rarely (4%) incorporate intraspecific trait variation (Saatkamp et al. 2019; Green et al. 2022) (but see Fricke, Tewksbury, and Rogers 2019). Further, germination does not appear to be constrained by evolutionary processes (Rogers et al. 2021). In some cases, however, closely related plant species have more similar germination success rates than more distantly related species (Wang et al. 2021; Lovas‐Kiss et al. 2020). For seed dispersers, age (Kubitzki and Ziburski 1994; Anderson, Saldaña Rojas, and Flecker 2009), body size (Anderson, Saldaña Rojas, and Flecker 2009; King, Milicich, and Burns 2011) and sex (Mancilla‐Leytón, González‐Redondo, and Vicente 2013) can influence germination success. However, the effect of disperser traits on germination remains an important research gap (Green et al. 2022; Zwolak 2018).

In this study, we conduct experiments with wild and captive animals to quantify the strength of ecological and evolutionary factors on seed germination. Accounting for phylogenetic effects, we (1) quantify the relative importance of three mechanisms: (i) pulp removal, (ii) seed priming, and (iii) feces (nutrient fertilization and/or microbiome) on two seed germination outcomes: (i) germination success and (ii) time‐to‐germination, (2) determine whether these outcomes are dependent on disperser species, and (3) estimate how *individual* plant and animal functional traits mediate the effects of gut passage on germination. We examined these patterns in both wild and captive experimental settings. Experimental settings included different plant and lemur species due to logistical feasibility and ethical considerations.

We hypothesize that (1) all mechanisms increase germination success rates and decrease time‐to‐germination; (2) (a) germination varies by disperser species and (b) through long‐term coevolutionary dynamics, germination is constrained by evolutionary history (phylogeny) such that closely related species have more similar germination success than distantly related species; and (3) in addition to phylogenetic effects, individual plant and animal functional traits influence the effects of seed dispersal on germination through their impacts on the mechanisms noted above. We predict germination success rates will increase with seed size and disperser body weight. If the predicted associations of mechanisms and seed germination success rates are consistent in captive and wild settings, we interpret this to indicate deep co‐evolutionary relationships between plants and animals. If, however, the associations are not consistent between wild and captive settings, experimental setting is a more important driver of dispersal outcomes. Experimentally mapping individual plant and disperser traits to their ecological function (i.e., germination) can advance a predictive framework of population dynamics, including in scenarios of frugivore loss due to environmental change (Saatkamp et al. 2019; Aslan et al. 2019).

We use lemurs, Madagascar's primary seed dispersers and only endemic primates (Razafindratsima 2014), and their food plants as a system to test these hypotheses. As the endemic primates of Madagascar, lemurs have 50+ million years of coevolutionary history with their food plants and play a vital role in forest ecosystems as seed dispersers. However, anthropogenic pressures such as deforestation threaten 94% of extant lemurs (Schwitzer et al. 2014) and 63% of endemic trees (Beech et al. 2021) with extinction. Through seed dispersal, lemurs largely determine the spatial distribution of seeds, influencing all subsequent processes that affect recruitment (Razafindratsima et al. 2022). The effects of lemur seed dispersal are generally positive: germination success rates tend to be higher for lemur‐dispersed seeds and time‐to‐germination is usually shorter compared to control seeds (Ramananjato et al. 2020; Simmen et al. 2006; Moses and Semple 2011). Even so, some lemur‐plant dispersal interactions result in lower or unchanged germination success rates (DeSisto et al. 2020; Razafindratsima and Razafimahatratra 2010). To our knowledge, this is the first study to address the effects of lemur seed dispersal experimentally with both captive and wild lemurs to test the impacts of both evolutionary and ecological processes.

## 2 Materials and Methods

### 2.1 Data Collection

#### 2.1.1 Germination Experiments

**FIGURE 1:**
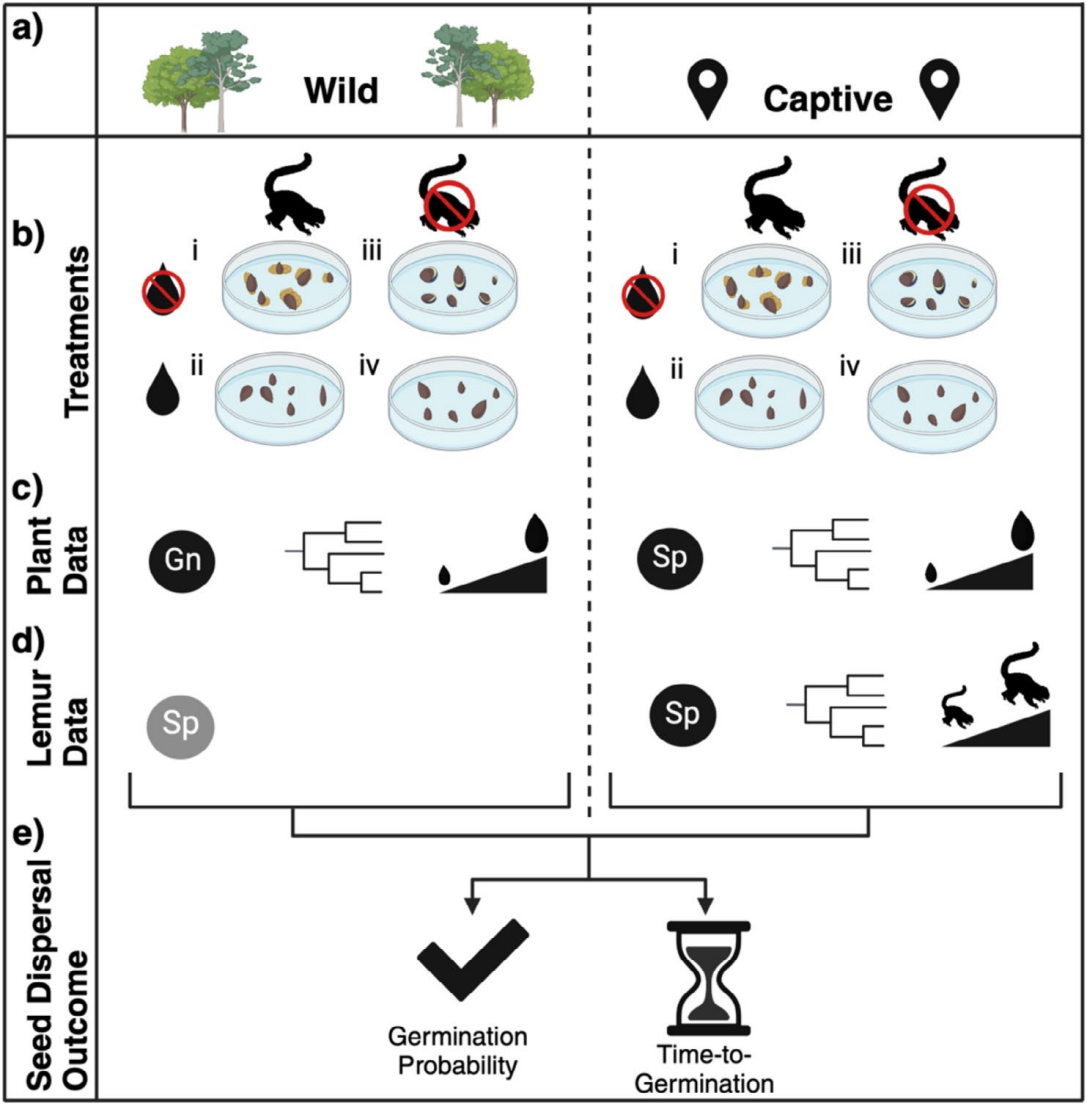
Schematic diagram representing the methods, including (a) environmental context (wild or captive), (b) the four experimental treatments, (c) plant data, (d) lemur data, and (e) seed dispersal outcomes (response variables, germination probability and time‐to‐germination). (c) Highlights that we considered plants to the genus level in the wild experiments but to the species level in the captive experiments; in both experimental settings, seed length was an individual‐level trait. (d) Highlights that, while we can only know species‐level lemur identities in some cases (wild lemurs were all genus *Eulemur*), we know species identity and individual‐level functional traits for all lemurs in the captive settings. Created with BioRender.

To assess the biological mechanisms that influence the effect of lemur gut passage on germination, we compared seed germination across four experimental treatments: (i) gut‐passed, unwashed, (ii) gut‐passed, manually washed to remove fecal material (feces removal), (iii) not gut‐passed, manually washed to remove pulp (pulp removal), and (iv) control, not gut‐passed, unwashed/ pulp not removed (Figure 1b). Compared to the control, treatment (iii) represents the effect of pulp removal and treatment (i) and (ii) represent the effects of pulp removal and potential seed priming caused by gut passage. Compared to treatment (ii), treatment (i) represents the effects of feces (potentially including nutrient fertilization and microbiome effects). Lemur‐passed and control seeds were randomly allocated to the four treatments. Processing included assignment to a treatment, measurement of seed length, and placement in a Petri dish with filter paper moistened with distilled water. Seeds assigned to washed treatments (ii and iv) were briefly rinsed with room‐temperature water until pulp/fecal matter was removed. We then monitored seeds for germination twice weekly for 100 days, adding distilled water to maintain moisture as necessary. We counted a seed as “germinated” at radicle emergence. All germinated seeds were removed from Petri dishes after data collection. Seeds that had not germinated by day 100 were recorded as having not germinated.

#### 2.1.2 Wild Animal Experiments

We conducted a field‐based germination experiment in Madagascar between June 2023 and May 2024. Fieldwork took place in the COMATSA‐Sud corridor in the SAVA (Sambava, Antalaha, Vohemar, Andapa) region of northeast Madagascar, a montane rainforest site. The World Wildlife Fund has co‐managed COMATSA with a decentralized system of local forest management associations since 2015, and the protected area is part of the *Complexe des Aires Prot'eg'ees d'Ambohimirahavavy‐Mariovorahona* (Goodman, Raherilalao, and Wohlhause 2019). Nine lemur species (*Eulemur albifrons*, *Eulemur rubriventer*, *Hapalemur occidentalis*, *Propithecus candidus*, *Allocebus trichotis*, *Cheirogaleus crossleyi*, *Microcebus lehilahytsara*, *Avahi laniger*, *Lepilemur saeli*) occur in the area.

We conducted surveys of diurnal lemurs on four 1‐km transects during the dry and wet seasons, searching for lemurs and lemur feces. Morning surveys were conducted at ∼07h00 and afternoon surveys at ∼13h00. When lemurs were encountered, we recorded species identity, group demographics, and geographic coordinates. After an encounter, we attempted to follow the group as long as possible to collect fecal samples. In the case of a defecation event, we collected feces, recording GPS coordinates and lemur species. We also opportunistically searched for lemur feces on the forest floor while conducting transect walks, identifying the lemur disperser to the genus level based on fecal morphology. We extracted and identified all seeds in the feces within 24 h of defecation. Seeds were identified to the vernacular name based on knowledge of the local community and later translated to Latin names based on herbarium specimens of leaves. Seeds were identified to and analyzed at the genus level. We collected control seeds from fruits on trees or on the forest floor from multiple individuals.

We collected feces with seeds from three lemur species (*Eulemur rubriventer*, *Eulemur albifrons*, *Propithecus candidus*), but 99% of fecal samples were obtained from *E. rubriventer* and *E. albifrons* . *P. candidus* is known to be a seed predator rather than disperser (Patel 2014), so we only analyzed samples from the two *Eulemur* species. In our experiments, we included seeds from plant genera for which we were able to collect sufficient data in terms of both lemur‐dispersed seeds (100 minimum) and control seeds (20 minimum): *Syzygium*, *Grewia*, *Cryptocarya*, *Breonadia*, *Pandanus*, and *Sterculia*. These plants are all large trees found in moist tropical forests. The genera are not endemic to Madagascar, but all except for *Breonadia* include species that are endemic to Madagascar (Tropicos.org 2024). For mean plant seed lengths, see Table S1.

#### 2.1.3 Captive Animal Experiments

We repeated an identical germination experiment to the wild animal study, replicating the four treatments in captive conditions at the Duke Lemur Center (DLC) in North Carolina, USA, from January–October 2023 (Figure 1). We conducted captive feeding trials and germination experiments with 8 lemur species ( *Varecia variegata variegata* , *Varecia rubra* , *Eulemur flavifrons* , *Eulemur coronatus* , *Eulemur mongoz* , *Microcebus murinus* , *Cheirogaleus medius* , and *Lemur catta* ). It is noteworthy that *E. flavifrons* , *E. coronatus* , and *E. mongoz* are in the same genus as the dispersers in the wild animal experiments. All captive lemur species are, however, different from those in the wild experimental setting due to species availability at the DLC.

Each lemur species was fed 6–10 common agricultural plant species as target species for the germination experiments: bell pepper ( *Capsicum annuum* ), cantaloupe ( *Cucumis melo* var. *cantalupensis*), tomato ( *Solanum lycopersicum* ), kiwi ( *Actinidia deliciosa* ), dragonfruit (*Selenicereus undatus*), honeydew melon ( *Cucumis melo* L.), butternut squash ( *Cucurbita moschata* ), blackberry ( *Rubus fruticosus* ), apple ( *Malus pumila* ), and cranberry (*Oxycoccus macrocarpus*). DLC feeding regulations and logistics of exporting fruits from Madagascar prohibited feeding captive lemurs exported fruits from Madagascar. Experiments consisted of 2–14 lemur individuals per species, depending on availability and logistics, for a total of 55 individuals (mean individuals per species = 6.8, SD = 3.7). Individual data on sex, age, and body weight of each animal were provided by the DLC. We conducted feeding trials in the morning and collected fecal samples for several hours after feedings. In cases where multiple animals were housed in one cage, we used colored dye to help identify fecal samples to the individual level.

Our analytical framework (Figure 1) examined the mechanisms by which lemur gut passage affects seed germination by investigating the effects of experimental treatments, species, and functional traits. Individual seed was always used as the replicates with germination success and time‐to‐germination as response variables. First, we combined all seeds to test the effects of wild and captive experiments. Second, we analyzed wild and captive experiments separately. We included phylogeny to account for non‐independence of species/ genera and estimate phylogenetic signal of responses. Time‐to‐germination could fit within a survival analysis framework; however, because we did not need to censor the data, we chose to use a similar MCMC (Markov chain Monte Carlo) framework as the germination success models. All analyses were conducted using R Version 4.3.1 (R Core Team 2023).

### 2.2 Statistical Analyses

#### 2.2.1 Wild and Captive Animal Experiments Combined

First, we tested the effects of treatment on germination success rate and time‐to‐germination for each seed. We pooled the wild and captive animal experiment data and conducted a logistic regression with germination as the binary response variable and a linear regression with time‐to‐germination (days, log‐transformed) as the response variable. To test whether the effects of treatments on germination success rate and time‐to‐germination differed in the wild compared to captive animal experiments, we included data type (wild or captive) as a factor. We constructed models in the R package lme4 (Bates et al. 2015), including treatment, data type, and their interaction as independent variables in the models.

#### 2.2.2 Wild Animal Experiments

We analyzed the effect of the four treatments on germination in the field by building a MCMC generalized linear mixed effects logistic regression with germination success as the dependent variable and a MCMC mixed effects linear regression with (log transformed) time‐to‐germination as the dependent variable. We treated the binary outcome, germination, as the dependent variable and treatment as the independent variable. Plant phylogeny and, when possible, lemur species were included as random effects. We also tested the effects of plant trait (seed size) variation on germination outcomes. To assess the effects of seed length on germination, we included individual seed length, treatment, and their interaction as fixed effects, with plant phylogeny, and, when possible, lemur species, as random effects. We were unable to assess the impacts of disperser traits in the wild dataset because collection of individual traits was not logistically possible in this study.

We used a logistic mixed effects model in a Bayesian framework (MCMCglmm package) (Hadfield 2010). For the prior parameters in all models, we assumed a multivariate normal distribution with mean 0 for fixed effects and inverse‐Wishart distributions (*V* = 1, *ν* = 0.002) for random effects. These are diffuse priors that are not overly informative and allow for exploring a wide parameter space. For each model, we ran the MCMC (Markov Chain Monte Carlo) algorithm for a total of 15,000 iterations. To ensure that the Markov chain reached stationarity, we discarded the first 1500 iterations as burn‐in. We did not apply any thinning, such that every iteration post‐burn‐in was retained for analysis. Following best practices for assessing MCMC results, we inspected trace plots of parameter values over generations to check for stabilization. We also ran the Gelman‐Rubin diagnostic using the package coda (Plummer et al. 2006) to ensure that the potential scale reduction factors were close to 1.

We accounted for plant phylogeny by incorporating the inverse phylogenetic relatedness matrices into the model. We trimmed the global angiosperm phylogeny (Smith and Brown 2018) to include only the genera in our dataset using V.PhyloMaker (Jin and Qian 2019). In addition to estimating the effects of treatment on germination probability, we estimated *λ*—phylogenetic signal describing the degree to which closely related taxa resemble one another following the Brownian motion model of evolution—to determine the phylogenetic signal in the residuals of the model.

#### 2.2.3 Captive Animal Experiments

We repeated the wild animal experiment treatment and seed size models for the captive lemur seed dispersal experiments. Because we identified lemurs to the individual level in the captive experiments, we could include the individual lemur identification nested within species as a random effect in the treatment and plant trait models. We could also test the effects of individual lemur traits on germination success and time‐to‐germination. In separate models, we determined the effects of variation in lemur traits by including individual body weight, sex, age, and activity pattern (diurnal or nocturnal) as independent variables; we also accounted for treatment as a dependent variable, and lemur and plant phylogeny as random effects. Continuous traits were transformed to the z‐score distribution. Species‐level plant and lemur phylogenies were included as random effects. For these models, we assessed the effects of plant species, rather than genera because we were able to identify all fruits and seeds to the species level. We detail our approach to lemur phylogeny in the Supporting Information (Table S5).

## 3 Results

### 3.1 Wild and Captive Animal Experiments Combined

In our wild animal experiments, we collected data from 3830 seeds passed by two lemur species ( *Eulemur rubriventer* and *Eulemur albifrons* ) and 1362 control seeds, for a total of 5192 seeds. We conducted subsequent analyses using six plant genera (*Syzygium*, *Grewia*, *Cryptocarya*, *Breonadia*, *Pandanus*, *Sterculia*) for which we were able to collect sufficient data both in terms of lemur‐dispersed and control seeds, for a total of 2130 seeds analyzed. In our captive animal experiments, we analyzed data collected from 5476 lemur‐passed and 642 control seeds, for a total of 6118 seeds across 10 readily available US food plant varieties (across nine species) and eight lemur species. Combining the wild and captive data, we analyzed 8248 seeds.

**FIGURE 2:**
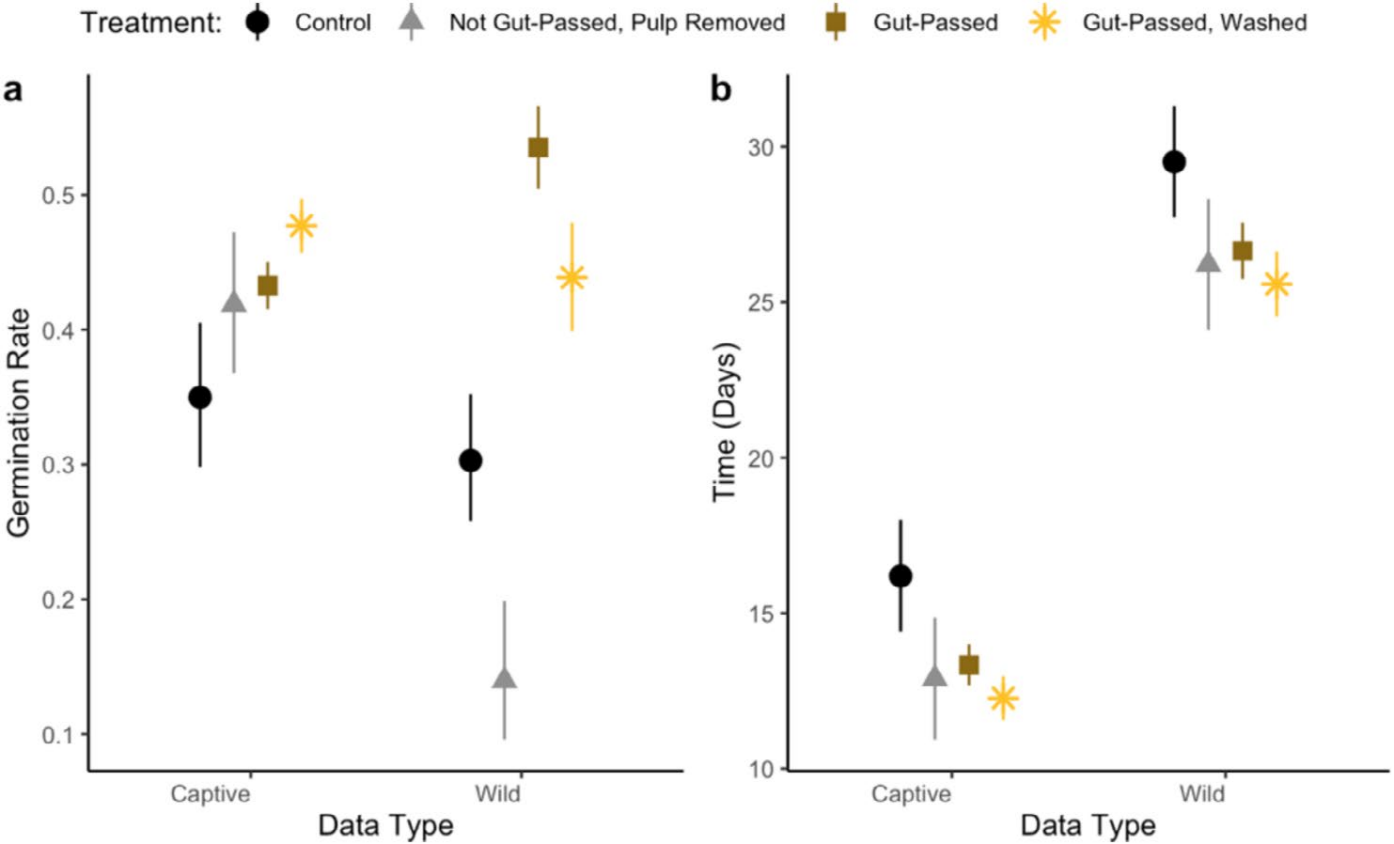
The effect of treatment on (a) germination success rate and (b) mean time‐to‐ germination for the wild and captive animal experiments combined. Points represent estimates and bars represent 95% confidence intervals.

Odds of germinating were 1.4 times greater for gut‐passed seeds (estimate = 0.349, *p* = 0.006, df = 8246) and 1.7 times greater for feces‐removed seeds (estimate = 0.529, *p* < 0.001) compared to control seeds (Figure 2a, Table S2). Seeds that were not gut‐passed and where the pulp was removed were 1.3 times more likely to germinate than control seeds (estimate = 0.292, *p* = 0.073, marginally significant). There was an interaction between experimental setting and treatment on germination probability; in other words, the influence of treatment on germination probability was stronger in the wild compared to captive setting (Table S2). Gut‐passed seeds also tended to germinate more quickly than seeds not passed by lemurs; gut‐passed seeds germinated 2.9 days before control seeds (*p* = 0.002, df = 3568) and feces‐removed seeds germinated 3.9 days before control seeds (*p* < 0.001; Figure 2b, Table S2). While wild seeds germinated 13.3 days slower than seeds in the captive experiment (*p* < 0.001), there was not a significant interaction between experimental setting and treatment on time‐to‐germination.

Different plant and animal species were used in the wild and captive experiments. Mean seed length in the wild (mean = 13.2 mm, SD = 28.3 mm) animal experiment was greater than the captive animal experiment (mean = 5.1 mm, SD = 3.6 mm; *t* = 13.121, *p* < 0.001, df = 5631.6; Table S1). Using species‐level body mass for wild lemurs (Razafindratsima, Yacoby, and Park 2018) and individual‐level mass for captive lemurs, average captive lemur mass was significantly lower than the mass of the wild species (*t* = −5.372, *p* < 0.001, df = 53.882). Due to these differences and environmental factors that could influence results, we conducted analyses treating the wild and captive experiments separately.

### 3.2 Wild Animal Experiments

#### 3.2.1 Treatment

**FIGURE 3:**
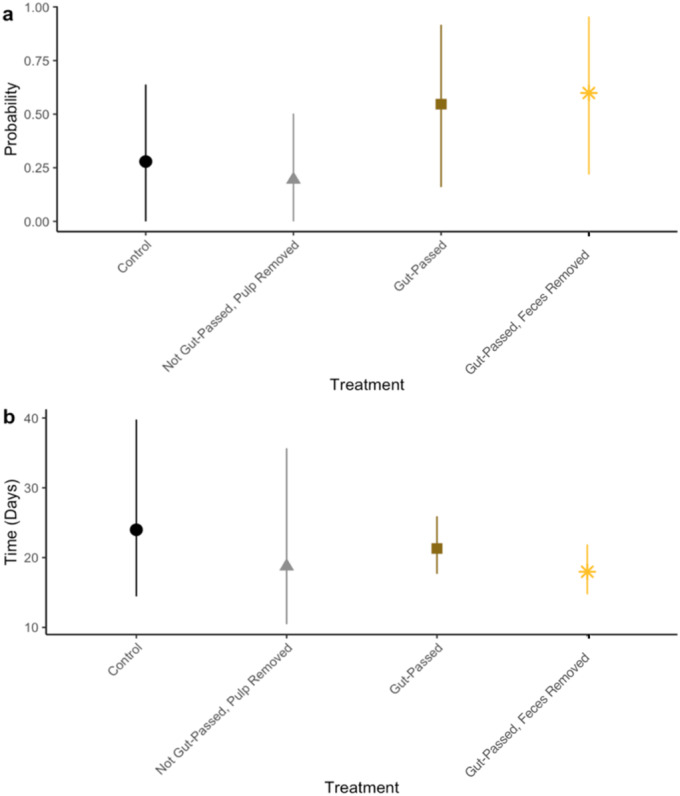
The effect of treatment on (a) germination probability and (b) mean time‐to‐germination for the wild animal experiments. Points represent estimates and bars represent 95% confidence intervals.

Controlling for plant phylogeny and lemur species as random effects, gut‐passed seeds were 5 times more likely to germinate than control seeds in experiments with wild lemurs (estimate = 1.614, *p* = 0.020; Figure 3a, Table S3). Feces‐removed seeds were 4 times more likely to germinate than control seeds (estimate = 1.385, *p* = 0.014), whereas removing pulp from the non‐passed seeds reduced the odds of germinating by 43% (estimate = −0.556, *p* = 0.076, marginally significant). Control seeds had higher mean germination probability (28% mean germination) compared to non‐gut‐passed pulp removed seeds (19%), although there was no statistically significant difference (Figure 3a). Feces‐removed seeds did not significantly differ in germination success rates (60%) compared to gut‐passed unwashed seeds (55%, Figure 3a). We detected a strong effect of plant phylogenetic effects, suggesting important variation in germination success across plant genera (*λ* = 0.88, 95% CI = 0.75–0.99).

Treatment did not affect time‐to‐germination in the wild animal experiments (Table S3). Compared to the control treatment's mean time‐to‐germination of 24.1 days, there was no significant difference in mean time‐to‐germination compared to the pulp removal (18.9 days), gut‐ passed unwashed (21.3 days), or feces removal treatments (18.0 days, Figure 3b, Table S3). Mean *λ* was 0.28 (0.03–0.61), suggesting a low plant phylogenetic effect on time‐to‐germination.

#### 3.2.2 Traits

**FIGURE 4:**
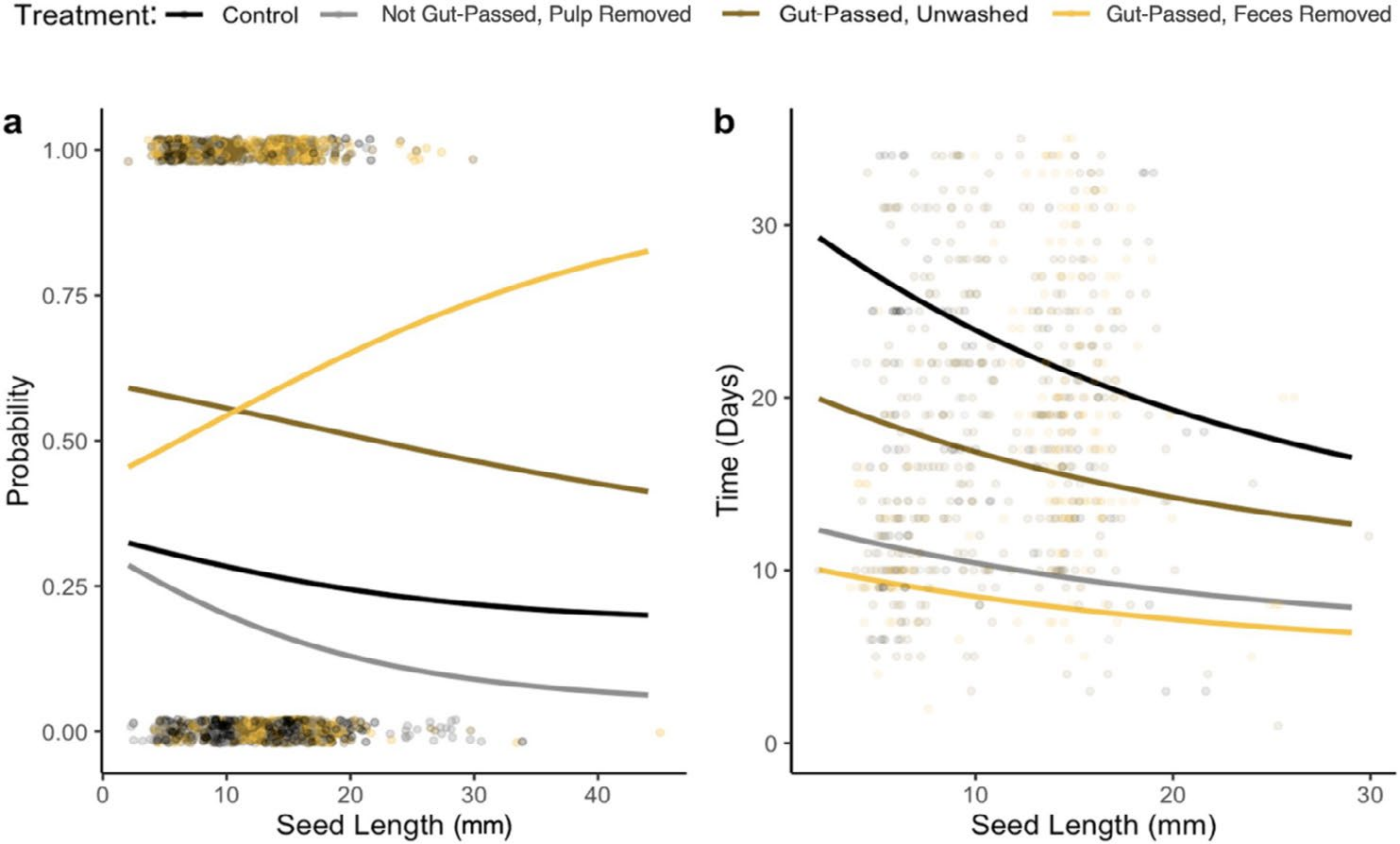
The interaction effect of treatment and individual variation in seed length on (a) germination probability and (b) time‐to‐germination in wild animal experiments. Points represent raw data and are jittered by 0.02 vertically to allow for visualization. In panel (b), points with time‐to‐germination > 35 days were excluded to improve ease of visualization.

Seed size was not significantly related to germination probability in the wild animal experiments (estimate = −0.031, *p* = 0.416, Figure 4a, Table S4). However, treatment influenced the effect of seed size on germination success rates. Compared to control seeds, the effect of seed size on germination probability was stronger for feces‐removed seeds (estimate = 0.088, *p* = 0.047). Controlling for the effects of seed length and its interaction with treatment, for each 1 mm increase in seed length, the odds of germination increased by 9.2% when feces were removed from the seeds. Plant phylogenetic signal was low: *λ* = 0.28 (0.03–0.61; Figure S1).

Overall, seed length was negatively, but not significantly, associated with time‐to‐germination (estimate = −0.025, *p* = 0.247; Figure 4b, Table S4). However, treatment influenced the effect of seed length on time‐to‐germination. Compared to control seeds, the effect of seed length on time‐to‐germination was more positive for non‐gut‐passed pulp removed seeds (estimate = 0.066, *p* = 0.009). Compared to control seeds, seeds germinated more quickly if the pulp (estimate = −0.978, *p* = 0.059, marginally significant) and feces (estimate = −1.057, *p* = 0.020) were removed. Plant phylogenetic effects were low (*λ* = 0.24, CI = 0.00–0.55).

### 3.3 Captive Animal Experiments

#### 3.3.1 Treatment

**FIGURE 5:**
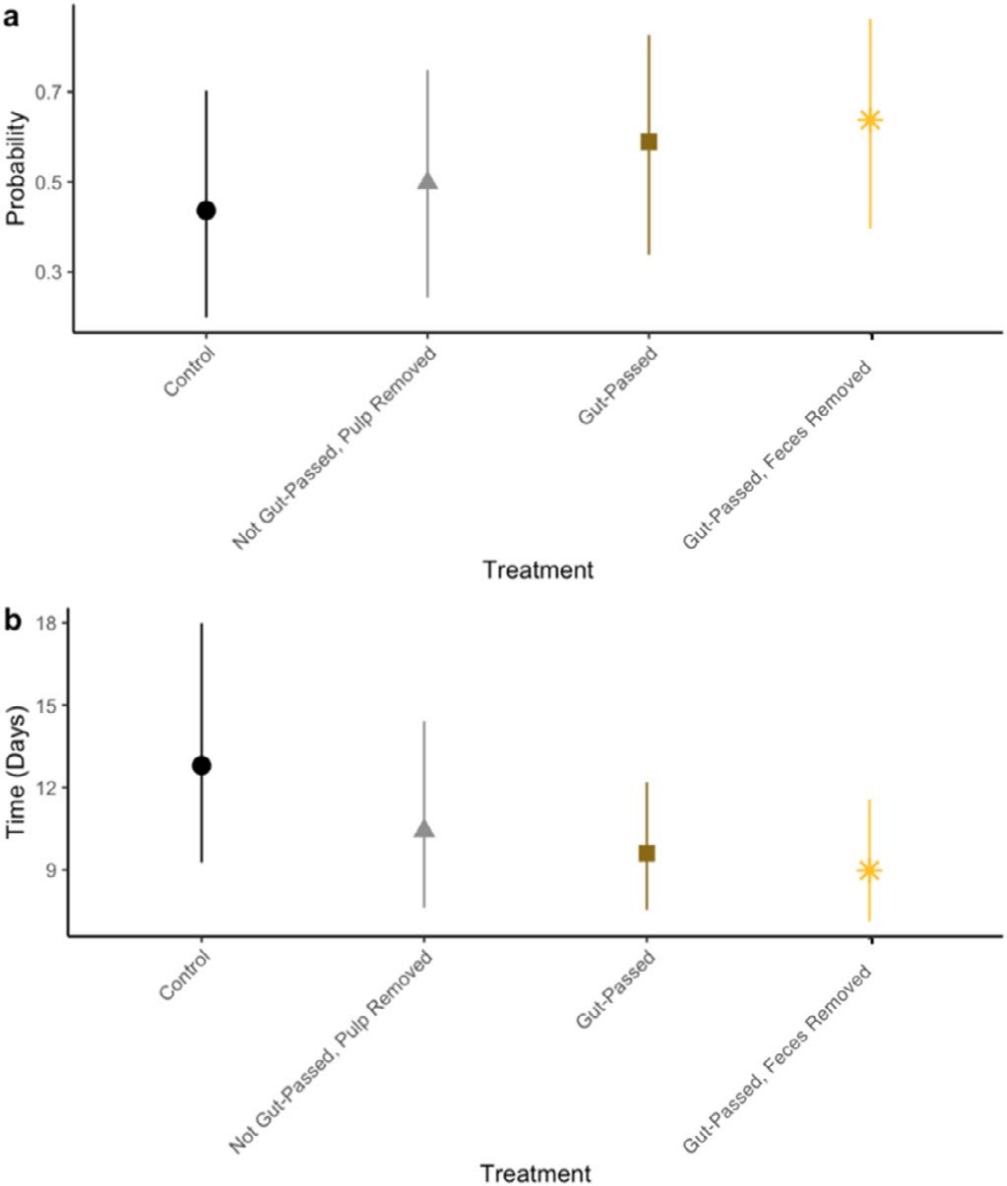
The effect of treatment on (a) germination success rate, and (b) mean time‐to‐germination for the captive animal experiments. Points represent estimates and bars represent 95% confidence intervals.

Captive lemur gut passage increased germination probability. Compared to control seeds, gut‐passed unwashed seeds were 2.4 times more likely to germinate (estimate = 0.922, *p* = 0.003; Figure 5a, Table S6) and feces‐removed seeds were 3.4 times more likely to germinate (estimate = 1.236, *p* < 0.001). Washing alone did not improve germination probability (estimate = 0.364, *p* = 0.305). We detected strong plant phylogenetic effects on germination probability (*λ* = 0.71, CI = 0.50–0.92).

Both unwashed gut‐passed seeds (9.6 days, *p* = 0.027) and feces‐removed seeds (9.0 days, *p* = 0.011) germinated more quickly than the control treatment's mean time‐to‐germination of 12.8 days. Mean time‐to‐germination was not significantly different from the pulp removal treatment (10.4 days, *p* = 0.117; Figure 5c, Table S6). There was low plant phylogenetic signal (*λ* = 0.27, CI = 0.08–0.48).

#### 3.3.2 Lemur Species

**FIGURE 6:**
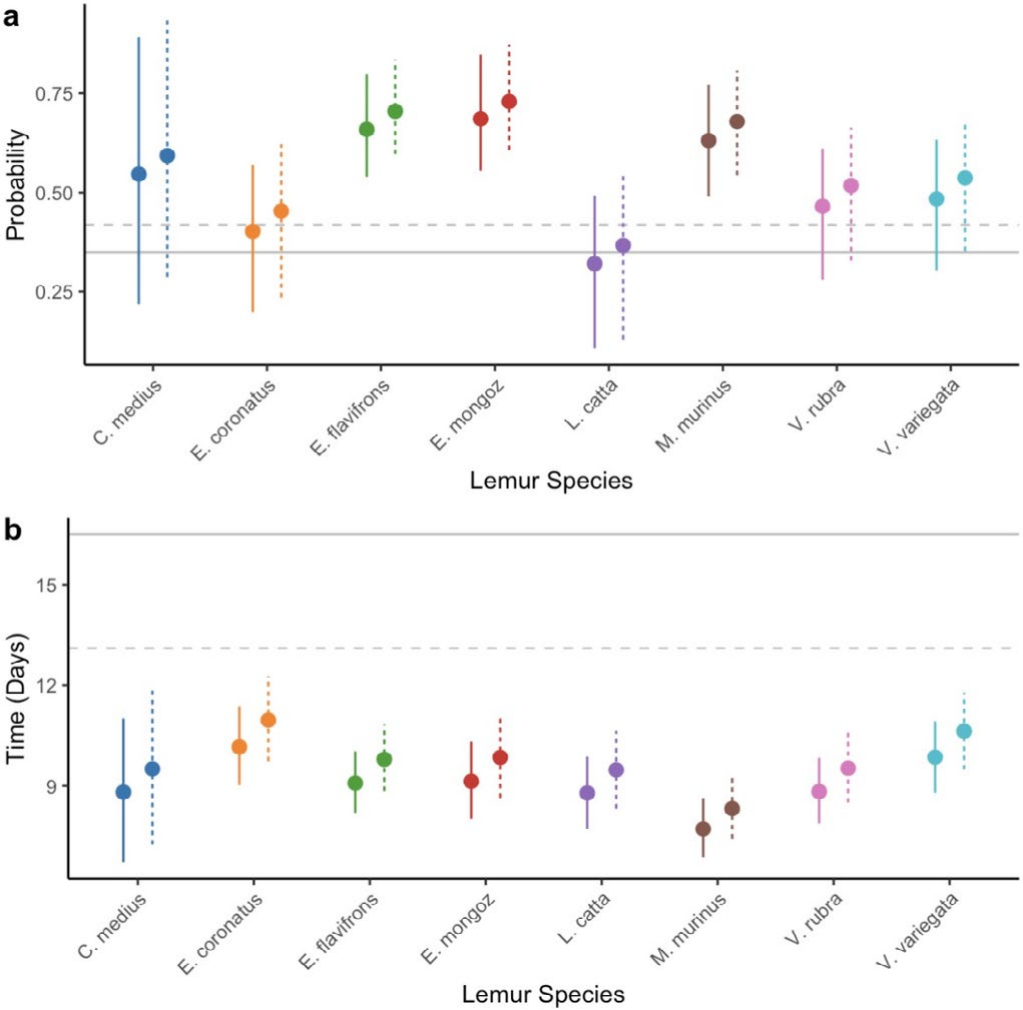
The effect of lemur species on (a) germination success rate, and (b) mean time‐to‐ germination for the captive animal experiments. Points represent estimates and bars represent 95% confidence intervals. Dashed lines represent the feces removal treatment, and solid lines represent the unwashed treatment. Horizontal lines represent mean germination success rate and mean time‐to‐germination of control seeds. In panel (c), the solid line represents observed values, and the shaded area represents the 95% confidence intervals.

Lemur species tended to have similar effects on germination probability and time‐to‐germination (Figure 6a,b, Table S7). However, *E. coronatus* (mean posterior probability in feces removal treatment = 49%, unwashed = 44%) and *L. catta* (42%/37%) had lower germination success rates than many other species (Figure 5a). Seeds passed by *E. mongoz* had the highest germination success rates (70%/66%), although they were not significantly more likely to germinate than those passed by many other lemur species. *λ* for plants was 0.81 (CI = 0.64–0.96).

Seeds passed by *E. coronatus* and *V. variegata* tended to take longer to germinate (11.0/10.2 and 10.6/9.9 days, depending on treatment), while those passed by *M. murinus* tended to germinate more quickly (8.3/7.7 days, depending on treatment; Figure 5b, Table S7).

#### 3.3.3 Traits

**FIGURE 7:**
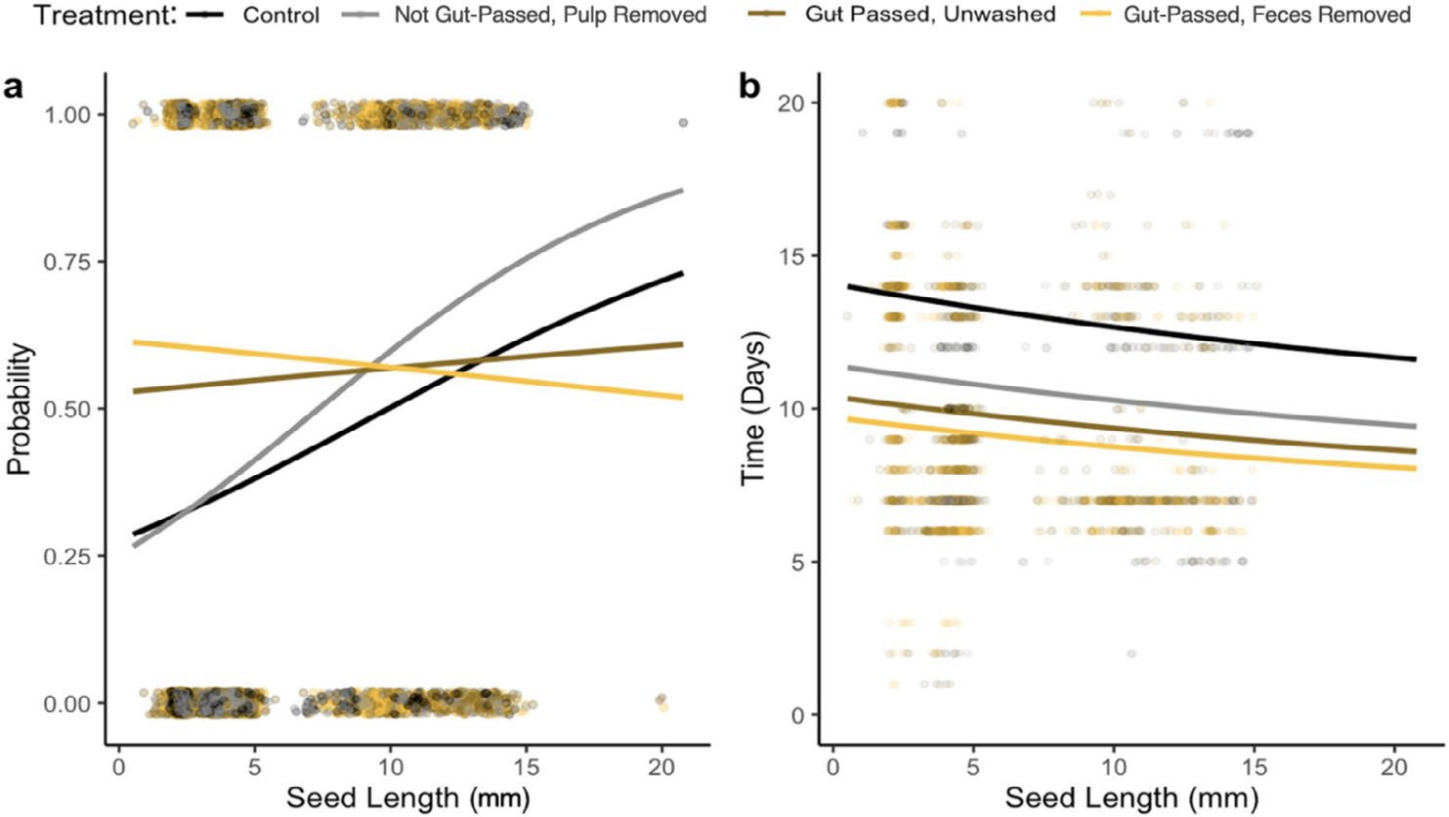
The interaction effect of treatment and individual variation in seed length on (a) germination probability and (b) time‐to‐germination in captive animal experiments. Points represent raw data and are jittered by 0.02 vertically to allow for visualization. In panel (b), points with time‐to‐germination > 20 days were excluded to improve ease of visualization.

Seed size was positively related to germination success rate; for every additional 1 mm in length, germination odds increased by 11.6% (estimate = 0.11 *p* = 0.019; Figure 7a). Treatment influenced the effect of seed size on germination success rates; compared to control seeds, the effect of seed size was weaker for gut‐passed unwashed seeds (estimate = −0.094, *p* = 0.019; Figure 7a, Table S8) and feces‐removed seeds (estimate = −0.132, *p* = 0.001). Controlling for the effects of seed length and its interaction with treatment, seeds were 3.4 times more likely to germinate if they were gut‐passed and unwashed (estimate = 1.230, *p* = 0.020) and 5.0 times more likely if feces were removed (estimate = 1.613, *p* = 0.004), compared to control seeds. *λ* for plants was high (0.86, CI = 0.75–0.97; Figure S2).

We did not detect evidence that treatment influenced the effect of seed size on time‐to‐ germination, so we proceeded with a model without the interaction term (Table S8). Seed length was negatively but not significantly associated with time‐to‐germination (estimate = −0.010, *p* = 0.509). *λ* for plants was low (0.27, CI = 0.11–0.54).

**FIGURE 8:**
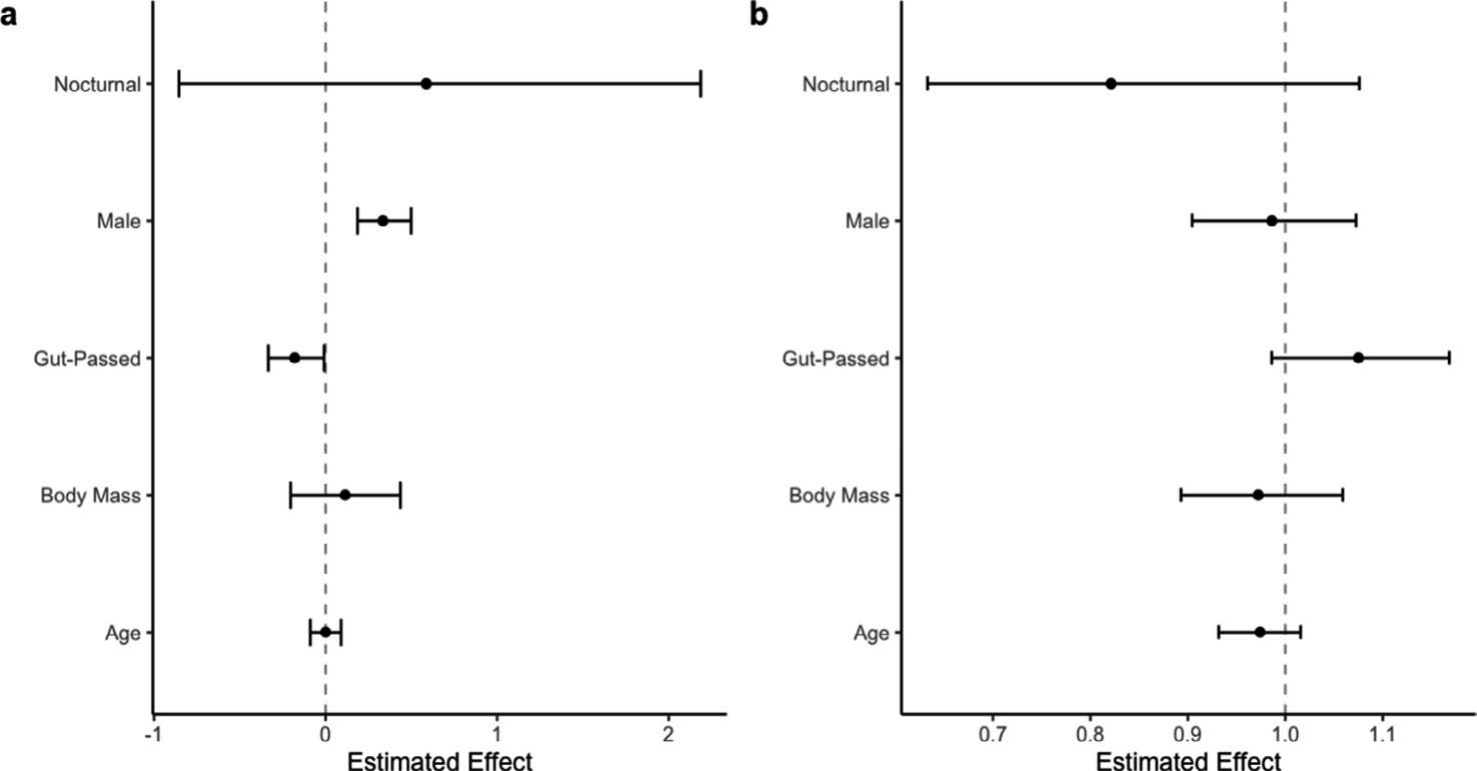
The effect of disperser traits on (a) germination success rate (log‐odds scale) and. (b) Time‐to‐germination in the captive animal experiments. Points represent model estimates and bars represent 95% confidence intervals. “Gut‐Passed” represents the feces removal treatment compared to the gut‐passed treatment without washing. Continuous variables mass and age were transformed to the *z*‐sore distribution.

Seeds passed by male lemurs were 39% more likely to germinate than seeds passed by females (estimate = 0.326, *p* < 0.001; Figure 8a, Table S9). No other lemur traits were significantly related to germination probability. We detected high plant phylogenetic signal (*λ* = 0.77, CI = 0.56–0.95) and low lemur phylogenetic signal (*λ* = 0.10, CI = 0.01–0.28) on germination success rate. No lemur traits were significantly associated with time‐to‐germination (Figure 8b, Table S9), and *λ* values were lower than those in the germination probability model for both plants (*λ* = 0.24, CI = 0.07–0.46) and lemurs (*λ* = 0.01, CI = 0.00–0.03).

## 4 Discussion

In this first study of both the ecological and evolutionary processes affecting the germination success and time of lemur dispersed seeds, we determined that gut passage by lemurs enhanced seed germination success rates in both wild and captive settings (Figures 3 and 5). Gut passage also reduced time‐to‐germination, but only in the captive experiments. We detected some disparities in germination outcomes based on the species of dispersers (Figure 6), and both individual seed length and the sex of individual dispersers was related to germination success (Figure 8). Although we detected strong plant phylogenetic effects in the germination probability models, the phylogenetic signal was low in time‐to‐germination models.

### 4.1 Mechanisms of Gut Passage Effects on Germination

Gut passage by frugivores typically promotes seed germination success rates (Rogers et al. 2021; Fuzessy et al. 2016). Our results support this pattern in both captive and wild settings (Figures 2a and 3a). Because both seed priming and pulp removal are intrinsic components of gut passage, it is difficult to separate their mechanistic effects on germination. Unlike previous research (Rogers et al. 2021), we did not identify a significant effect of pulp removal on germination success rates (Figures 3a and 5a). Compared with control seeds, germination rate was slightly higher when pulp was removed in the captive animal experiments but lower in the wild animal experiments, but results were not statistically significant. Our results, therefore, suggest that the mechanism by which lemur seed dispersal promotes germination is likely seed priming, physical or chemical changes to the seeds during gut passage. Additionally, fecal material may have prevented germination (Figures 3 and 5), possibly by promoting the growth of pathogens such as mold, which we observed on seeds in our experiments. Frugivores can disperse seeds and their associated arbuscular mycorrhizal fungi (Correia et al. 2019), but further research should explore the extent to which frugivores, including lemurs, disperse pathogenic fungi. Future research should also investigate the effect of lemur gut passage on the viability of seeds that did not germinate.

Gut passage increased germination probability more in wild compared to captive experimental settings, likely due to species differences. Plant identity explained much more variance in germination than disperser species, emphasizing the importance of plant identity for germination outcomes. All plants in captivity were cultivated, and there may have been artificial selection to increase germination in the absence of gut passage in these species. There also could be artificial selection for seed size (Figures S1 and S2). Wild plant species in Madagascar may have co‐evolved with lemurs such that lemur seed dispersal mutualism is more effective in promoting germination of plants native to Madagascar. There is evidence, however, that lemur‐plant coevolution may be weak (Fuzessy et al. 2023), and plant morphological traits in Madagascar are more likely driven by abiotic conditions than lemur frugivory (Bollen et al. 2005). Another explanation could be differences in the gut microbiome; gut microbiome diversity is often lower in captive primates than wild primates (Bornbusch et al. 2022; Frankel et al. 2019; McKenzie et al. 2017), and housing conditions affect microbial communities in captive lemur feces (Bornbusch et al. 2022). The role of the microbiome in influencing germination remains unclear (Nelson 2018).

Time‐to‐germination affects components of plant fitness such as fecundity and survival (Donohue et al. 2010). Similar to other primates (Rogers et al. 2021; Fuzessy et al. 2016), seeds passed by captive lemurs germinated significantly faster than control seeds (Figure 5), suggesting that gut passage may confer a competitive advantage. Previous studies found pulp removal to be the primary mechanism through which gut passage affects germination (Fricke et al. 2019; Rogers et al. 2021). Our results, however, suggest that seed priming during gut passage, but not pulp removal or feces effects, likely decreased time‐to‐germination in captive settings (Figure 5). Gut passage did not affect time‐to‐germination in the wild experiments. This likely because captive seeds were from human food plants that have been bred for agriculture, unlike the wild seeds in Madagascar. Our results suggest that the wild seeds in our study may be adapted to have higher germination success, rather than time‐to‐germination, after lemur gut passage.

We detected strong plant phylogenetic signal on germination probability; closely related plants responded more similarly to treatments than more distantly related plants. These results support phylogenetic niche conservatism, illustrating a potential strong co‐evolutionary relationship between plants and lemurs in terms of germination success rates. Previous research on phylogenetic effects on seed germination has been mixed (Wang et al. 2021; Rogers et al. 2021). Nevertheless, plant phylogeny partially explains variation in seed survival in bird guts (Lovas‐Kiss et al. 2020), and phylogenetic congruence has been observed between Neotropical plants and their frugivore dispersers (Fuzessy et al. 2022). Plant phylogenetic effects on time‐to‐germination were weaker than those on germination probability, further suggesting that lemurs may not be adapted to have low germination times. Accounting for phylogenetic non‐independence is important for testing biologically meaningful hypotheses about germination success.

### 4.2 Interspecific Variation in Effects on Germination

Germination probability and time‐to‐germination of gut‐passed seeds tended to be consistent across different lemur species. This is likely because no lemur species are particularly well‐adapted to disperse agricultural fruits. Nocturnal lemurs had comparable seed germination to larger‐bodied, primarily frugivorous lemur species such as *V. variegata* and *E. flavifrons* . Seeds dispersed by *M. murinus* , a small‐bodied nocturnal lemur, also tended to have lower mean time‐to‐germination than those passed by other lemur species (Figure 6b). Although nocturnal lemurs are often overlooked as seed dispersers, they can perform this critical function for a wide variety of plant species (Ramananjato et al. 2020). Seeds passed by *E. coronatus* and *L. catta* had lower germination success rates than other species. Gut passage by wild *E. coronatus* has been shown to both negatively (Steffens 2020) and positively (Chen et al. 2015) affect germination success. In Madagascar, *L. catta* can disperse viable tamarind seeds (Mertl‐Millhollen et al. 2011), but its role in seed dispersal of native species is relatively unexplored. *L. catta* is classified as an opportunistic omnivore rather than a strict frugivore (Gould 2007), which could partially explain its lower seed dispersal effectiveness.

### 4.3 Plant and Frugivore Functional Traits Affect Germination

Plant morphological traits mediate the consequences of frugivory for plant reproduction and survival (Starrfelt and Kokko 2012; Rogers et al. 2021). While theory predicts that large‐seeded plants should germinate more quickly than small‐seeded plants, the opposite has been observed across tropical forests (Norden et al. 2009). In our wild experiments, large seeds were much more likely to germinate than small seeds in the feces removal treatment (Figure 4b). This suggests that seed length was important for mediating the effects of feces (nutrient fertilization/ microbiome) and deinhibition due to pulp removal on germination probability. In wild settings, de‐inhibitory effects may be more important for increasing germination probability in larger seeds due to their greater nutritional demands and thick seed coats (Traveset, Rodríguez‐Peérez, and Pías 2008). In captive experiments, however, smaller seeds tended to benefit more from gut passage than larger seeds (Figures 4 and 7). Smaller agricultural seeds may be more vulnerable to seed priming during gut passage due to thinner and more easily permeable seed coats. Additionally, domestication of agricultural plants could influence their susceptibility to the effects of gut passage; for example, some plant species (bell pepper, butternut squash, cantaloupe, honeydew melon, and tomato) are typically propagated through seed while others are typically propagated asexually (apple, blackberry, cranberry, dragonfruit, and kiwi). The relationships between plant traits and germination vary by disperser taxa; for example, in wild settings, gut passage by small‐to‐medium sized frugivorous birds is more beneficial for small seeds than large seeds (Fricke et al. 2019). Additionally, seed size is not significantly related to germination success rate or time after dispersal by Neotropical primates (Fuzessy et al. 2016). Seed length and germination outcomes were also shaped by evolutionary processes, potentially due to selection pressures conserved within lineages (Figures S1 and S2).

Disperser traits are commonly neglected in seed dispersal research (Zwolak 2018; Green et al. 2022). We overcame feasibility challenges associated with collecting intra‐specific disperser trait data through the captive animal experiment. Seeds passed by male lemurs were more likely to germinate than those passed by females (Figure 8a). While the mechanism of this relationship remains unknown, it could be due to sexual dimorphism in activity level, gut morphology, microbiome, and/ or hormone levels. Sex is seldom accounted for in seed dispersal research, but female rabbits are more likely to defecate intact seeds than male rabbits (Mancilla‐Leytón, González‐Redondo, and Vicente 2013) and female tortoises have longer gut retention times than male tortoises (Lautenschlager, Souza, and Galetti 2022). We did not detect significant relationships between germination probability and other lemur traits including mass, age, and activity pattern. These results are likely taxon‐specific; for example, they contrast evidence which suggests that seeds are more likely to survive gut passage in older fish than younger fish (Kubitzki and Ziburski 1994; Anderson, Saldaña Rojas, and Flecker 2009) and that larger‐bodied insects and fish confer more positive effects on seed dispersal quality than smaller‐bodied insects and fish (Anderson, Saldaña Rojas, and Flecker 2009; King, Milicich, and Burns 2011; Correa et al. 2015). Our results emphasize that accounting for individual plant and disperser traits, as well as species and phylogenetic effects, is important for advancing a mechanistic understanding of the ecological and evolutionary consequences of seed dispersal.

## Author Contributions

**Camille M. M. DeSisto:** conceptualization (lead), data curation (lead), formal analysis (lead), funding acquisition (lead), investigation (equal), methodology (lead), project administration (lead), visualization (lead), writing – original draft (lead), writing – review and editing (lead). **Zico Zandry:** investigation (equal), methodology (supporting), project administration (supporting), writing – review and editing (supporting). **Telesy Feno:** investigation (equal), methodology (supporting), project administration (supporting), writing – review and editing (supporting). **Borna Zareiesafandabadi:** investigation (equal), writing – review and editing (supporting). **Jean Randrianasy:** investigation (equal), writing – review and editing (supporting). **Jean Tiamanana:** investigation (equal), writing – review and editing (supporting). **Dominique Randrianasolo:** investigation (equal), writing – review and editing (supporting). **Manadina Rasolofo:** investigation (equal), writing – review and editing (supporting). **George Raveloson:** investigation (equal), writing – review and editing (supporting). **Franclin Zerimanana:** investigation (equal), writing – review and editing (supporting). **Onja Razafindratsima:** conceptualization (supporting), writing – review and editing (supporting). **James P. Herrera:** conceptualization (supporting), funding acquisition (supporting), project administration (supporting), resources (supporting), supervision (equal), writing – review and editing (supporting). **John R. Poulsen:** conceptualization (supporting), formal analysis (supporting), supervision (equal), writing – review and editing (supporting).

## Conflicts of Interest

The authors declare no conflicts of interest.

## Supporting information


Appendix S1.

## Data Availability

Germination experiment data and code supporting the results are archived at https://gitfront.io/r/camilledesisto/aK6f9M7hFyYY/LemurGermination2. Lemur trait data are proprietary to the DLC and may be made available upon request.
