# Supplementary material for: Functional Traits and Phylogenetic Effects Drive Germination of Lemur‐Passed Seeds
Source: Ecol Evol. 2025 Feb 4;15(2):e70881. doi: 10.1002/ece3.70881 (PMC11794832; doi:10.1002/ece3.70881)
Supplement: Supplementary file 1 — Appendix S1. [file ECE3-15-e70881-s001.docx]

# Appendix

## Methods

Although we collected lemur species data for all gut-passed seeds, MCMCglmm cannot fit models where observations are missing from the phylogeny, i.e., in the case of control seeds which were not passed by a lemur species. We therefore tested the validity of conducting treatment models that included a lemur species but not lemur phylogenetic effects. Excluding control seeds, we conducted models testing the effects of treatment on germination success and time-to-germination, where a) plant phylogeny and lemur ID were nested within lemur species as random effects, and b) plant phylogeny and lemur ID nested within lemur phylogeny (Herrera & D´avalos, 2016) as random effects. The very low lemur *λ* values (0.04, CI = 0-0.2 for germination success; 0.01, CI = 0.0-0.2 for time-to-germination) in b models suggested that lemur phylogenetic signal in germination outcomes was very low (not different from 0), thus we included only lemur species as random effects in captive animal models, except for the lemur trait model which only included lemur-passes seeds.

## Figures


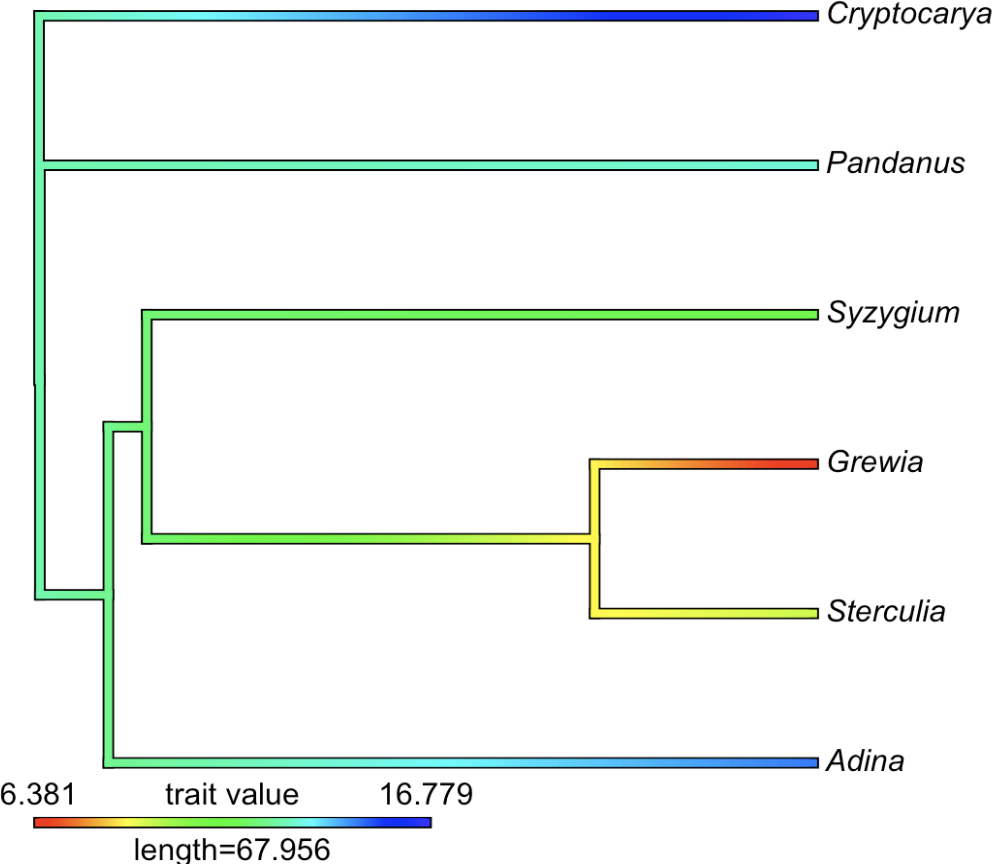


Figure S1: Phylogenetic tree of plant species in the wild animal experiment with continuous trait mapping of seed length. Branch colors represent mean length (mm) of plants. The tree was generated using the phytools package (Revell, 2012).


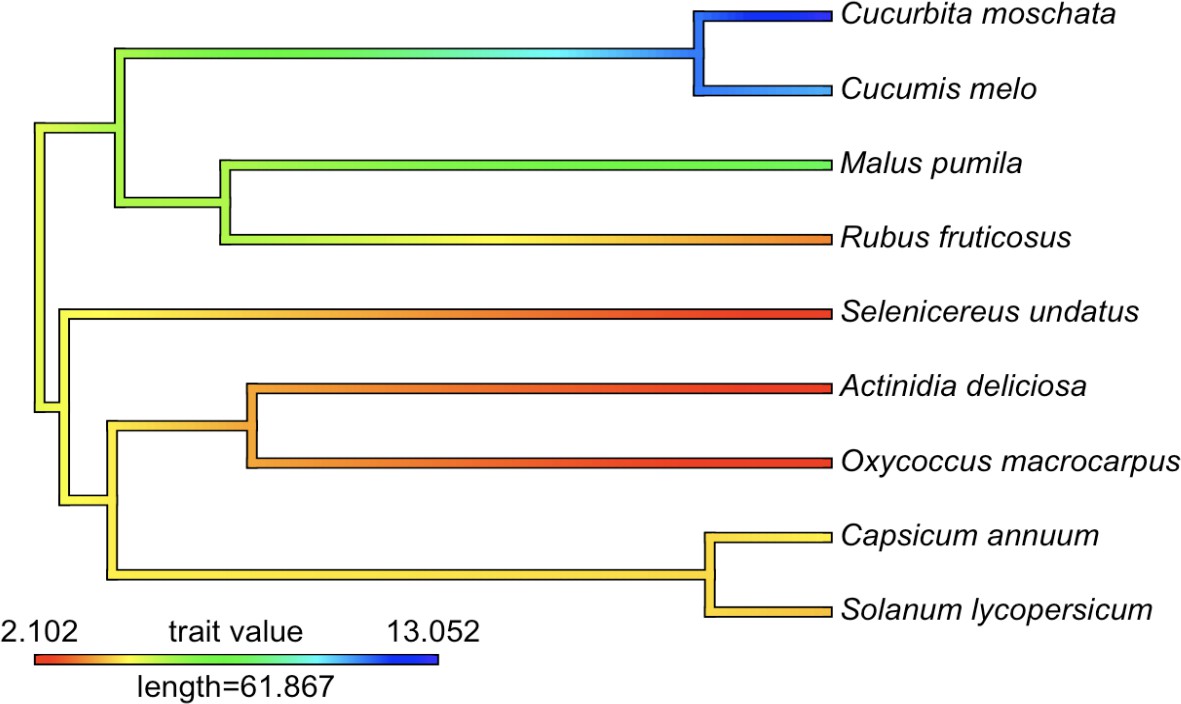


Figure S2: Phylogenetic tree of plant species in the captive animal experiment with continuous trait mapping of seed length. Branch colors represent mean length (mm) of plants. The tree was generated using the phytools package (Revell, 2012).

## Tables

| **Habitat** | **Plant** | **Mean**  **Seed Length (mm)** | **SD**  **Seed Length (mm)** |
| --- | --- | --- | --- |
|  | *Breonadia* | 15.5 | 2.3 |
|  | *Cryptocarya* | 15.5 | 2.5 |
| Wild | *Grewia*  *Pandanus* | 7.3  17.9 | 2.1  7 |
|  | *Sterculia* | 8.9 | 0.8 |
|  | *Syzygium* | 8.4 | 3.6 |
|  | *Actinidia deliciosa* | 2.3 | 0.2 |
|  | *Capsicum annuum* | 4.6 | 0.8 |
|  | *Cucumis melo L.* | 12.2 | 1.6 |
|  | *Cucumis melo var. cantalupensis* | 10 | 1.3 |
| Captive | *Cucurbita moschata*  *Malus pumila* | 13  8 | 1.1  0.9 |
|  | *Oxycoccus macrocarpus* | 2.1 | 0.3 |
|  | *Rubus fruticosus* | 3.4 | 0.4 |
|  | *Selenicereus undatus* | 2.2 | 0.2 |
|  | *Solanum lycopersicum* | 4 | 0.7 |

Table S1: Summary of the seed lengths of plants found in lemur feces in both the wild and captive experiments.

| **Outcome** | **Variable** | **Estimate** | **Std. Error** | **p-value** |
| --- | --- | --- | --- | --- |
|  | Intercept | -0.620 | 0.120 | *<*0.001*** |
|  | Not gut-passed, pulp removed | 0.292 | 0.163 | 0.073+ |
|  | Gut-passed, unwashed | 0.349 | 0.126 | 0.006** |
| Germination | Gut-passed, feces removed | 0.529 | 0.127 | *<*0.001*** |
| rate | Wild | -0.213 | 0.166 | 0.199 |
|  | Not gut-passed, pulp removed: Wild | -0.128 | 0.294 | *<*0.001*** |
|  | Gut-passed, unwashed: Wild | 0.625 | 0.181 | *<*0.001*** |
|  | Gut-passed, feces removed: Wild | 0.057 | 0.190 | 0.765 |
|  | Intercept | 16.195 | 0.919 | *<*0.001*** |
| Time-to- germination | Not gut-passed, pulp removed  Gut-passed, unwashed Gut-passed, feces removed | -3.303  -2.862  -3.933 | 1.341  0.938  0.963 | 0.014*  0.002**  *<*0.001*** |
|  | Wild | 13.324 | 0.499 | *<*0.001*** |

Table S2: Results for the treatment models in the wild experiments and captive experiments combined. *** indicates *p <* 0.001; ** indicates 0.001 *< p <* 0.01; * indicates 0.01 *< p <* 0.05, + indicates 0.05 *< p <* 0.1.

| **Outcome** | **Variable** | **Estimate** | **Lower CI** | **Upper CI** | **pMCMC** |
| --- | --- | --- | --- | --- | --- |
|  | Intercept | -1.156 | -3.32 | 0.975 | 0.228 |
| Germination | Not gut-passed, pulp removed | -0.556 | -1.227 | 0.042 | 0.078+ |
| Rate | Gut-Passed, Unwashed | 1.385 | 0.523 | 2.203 | 0.020* |
|  | Gut-passed, feces removed | 1.614 | 0.809 | 2.51 | 0.014* |
|  | Intercept | 3.183 | 2.674 | 3.714 | 0.001** |
| Time-to-Germination | Not gut-passed, pulp removed  Gut-Passed, Unwashed | -0.244  -0.123 | -0.672  -0.643 | 0.194  0.37 | 0.272  0.460 |
|  | Gut-passed, feces removed | -0.293 | -0.811 | 0.223 | 0.157 |

Table S3: Results for the treatment models in the wild experiments. *** indicates *p <* 0.001;

** indicates 0.001 *< p <* 0.01; * indicates 0.01 *< p <* 0.05, + indicates 0.05 *< p <* 0.1.

| **Outcome** | **Variable** | **Estimate** | **Lower CI** | **Upper CI** | **pMCMC** |
| --- | --- | --- | --- | --- | --- |
|  | Intercept | -0.838 | -3.217 | 1.347 | 0.416 |
|  | Length | -0.031 | -0.11 | 0.04 | 0.42 |
|  | Not gut-passed, pulp removed | -0.113 | -1.456 | 1.354 | 0.865 |
| Germination | Gut-Passed, Unwashed | 1.271 | 0.068 | 2.567 | 0.043* |
| Rate | Gut-passed, feces removed | 0.446 | -0.878 | 1.777 | 0.503 |
|  | Length: Not gut-passed, pulp removed | -0.036 | -0.147 | 0.067 | 0.515 |
|  | Length:Gut-Passed, Unwashed | 0.011 | -0.072 | 0.089 | 0.793 |
|  | Length:Gut-passed, feces removed | 0.088 | 0.003 | 0.173 | 0.047* |
|  | Intercept | 3.382 | 2.644 | 4.083 | 0.002** |
|  | Length | -0.025 | -0.067 | 0.017 | 0.247 |
|  | Not gut-passed, pulp removed | -0.978 | -2.006 | 0.036 | 0.059+ |
| Time-to- | Gut-Passed, Unwashed | -0.357 | -1.023 | 0.405 | 0.264 |
| Germination | Gut-passed, feces removed | -1.057 | -1.818 | -0.269 | 0.02* |
|  | Length: Not gut-passed, pulp removed | 0.071 | -0.018 | 0.157 | 0.114 |
|  | Length:Gut-Passed, Unwashed | 0.025 | -0.024 | 0.07 | 0.299 |
|  | Length:Gut-passed, feces removed | 0.066 | 0.015 | 0.116 | 0.009** |

Table S4: Results for the seed size models in the wild experiments. *** indicates *p <* 0.001;

** indicates 0.001 *< p <* 0.01; * indicates 0.01 *< p <* 0.05, + indicates 0.05 *< p <* 0.1.

| **Model Type** | **Variable** | **Estimate** | **Lower CI** | **Upper CI** | **pMCMC** |
| --- | --- | --- | --- | --- | --- |
| Lemur Phylogeny  Random Effect | Intercept  Gut-passed, feces removed | 0.160  -0.223 | -0.823  0.059 | 1.09  0.372 | 0.715  0.007** |
| Lemur Species  Random Effect | Intercept  Gut-passed, feces removed | 0.189  0.208 | -0.724  0.059 | 1.221  0.370 | 0.662  0.009** |

Table S5: Results for the treatment sub-models (including only lemur-passed seeds) in the captive experiments. *** indicates *p <* 0.001; ** indicates 0.001 *< p <* 0.01.

| **Outcome** | **Variable** | **Estimate** | **Lower CI** | **Upper CI** | **pMCMC** |
| --- | --- | --- | --- | --- | --- |
|  | Intercept | -0.376 | -2.005 | 1.196 | 0.611 |
| Germination Rate | Not gut-passed, pulp removed  Gut-Passed | 0.364  0.922 | -0.332  0.252 | 1.125  1.754 | 0.305  0.003** |
|  | Gut-passed, feces removed | 1.236 | 0.449 | 2.086 | *<*0.001*** |
|  | Intercept | 2.549 | 2.211 | 2.884 | *<*0.001*** |
| Time-to-Germination | Not gut-passed, pulp removed  Gut-Passed | -0.206  -0.288 | -0.461  -0.533 | 0.045  -0.019 | 0.117  0.027* |
|  | Gut-passed, feces removed | -0.355 | -0.613 | -0.100 | 0.011* |

Table S6: Results for the treatment models in the captive experiments. *** indicates *p <* 0.001; ** indicates 0.001 *< p <* 0.01; * indicates 0.01 *< p <* 0.05, + indicates 0.05 *< p <* 0.1.

| **Outcome** | **Variable** | **Esimate** | **Lower CI** | **Upper CI** | **pMCMC** |
| --- | --- | --- | --- | --- | --- |
|  | Intercept | 0.562 | -0.664 | 1.685 | 0.310 |
|  | Gut-Passed | -0.217 | -0.395 | -0.037 | 0.015* |
|  | *E. coronatus* | -0.608 | -1.086 | -0.121 | 0.011* |
| Germination Rate | *E. flavifrons*  *E. mongoz*  *L. catta* | 0.244  0.32  -0.887 | -0.182  -0.206  -1.4 | 0.657  0.826  -0.368 | 0.259  0.221  *<*0.001*** |
|  | *M. murinus* | 0.147 | -0.3 | 0.613 | 0.542 |
|  | *V. rubra* | -0.394 | -0.853 | 0.037 | 0.072* |
|  | *V. variegata* | -0.327 | -0.777 | 0.126 | 0.150 |
|  | Intercept | 2.245 | 2.01 | 2.486 | *<*0.001*** |
|  | Gut-Passed | -0.076 | -0.118 | -0.033 | 0.001** |
|  | *E. coronatus* | 0.148 | 0.028 | 0.264 | 0.013* |
|  | *E. flavifrons* | 0.036 | -0.068 | 0.133 | 0.486 |
| Time-to-Germination | *E. mongoz* | 0.041 | -0.087 | 0.167 | 0.531 |
|  | *L. catta* | 0.003 | -0.118 | 0.124 | 0.967 |
|  | *M. murinus* | -0.127 | -0.239 | -0.012 | 0.031* |
|  | *V. rubra* | 0.008 | -0.096 | 0.12 | 0.884 |
|  | *V. variegata* | 0.118 | 0.011 | 0.228 | 0.032* |

Table S7: Results for the lemur species models in the captive experiments. *** indicates *p*

*<* 0.001; ** indicates 0.001 *< p <* 0.01; * indicates 0.01 *< p <* 0.05, + indicates 0.05 *< p <*

0.1.

| **Outcome** | **Variable** | **Estimate** | **Lower CI** | **Upper CI** | **pMCMC** |
| --- | --- | --- | --- | --- | --- |
|  | Intercept | -1.103 | -2.611 | 0.358 | 0.139 |
|  | Length | 0.11 | 0.018 | 0.2 | 0.019* |
|  | Not gut-passed, pulp removed | -0.158 | -1.015 | 0.686 | 0.738 |
| Germination | Gut-Passed | 1.23 | 0.118 | 2.277 | 0.020* |
| Rate | Gut-passed, feces removed | 1.613 | 0.522 | 2.724 | 0.004** |
|  | Length: Not gut-passed, pulp removed | 0.06 | -0.045 | 0.165 | 0.261 |
|  | Length: Gut-Passed | -0.094 | -0.173 | -0.018 | 0.019* |
|  | Length: Gut-passed, feces removed | -0.132 | -0.211 | -0.055 | 0.001** |
|  | Intercept | 2.626 | 2.211 | 3.006 | *<*0.001** |
|  | Length | -0.01 | -0.04 | 0.021 | 0.509 |
| Time-to-Germination | Not gut-passed, pulp removed | -0.208 | -0.474 | 0.039 | 0.107 |
|  | Gut-Passed | -0.297 | -0.552 | -0.039 | 0.023* |
|  | Gut-passed, feces removed | -0.364 | -0.613 | -0.097 | 0.007** |

Table S8: Results for the seed size models in the captive experiments. *** indicates *p <* 0.001; ** indicates 0.001 *< p <* 0.01; * indicates 0.01 *< p <* 0.05, + indicates 0.05 *< p <* 0.1.

| **Outcome** | **Variable** | **Estimate** | **Lower CI** | **Upper CI** | **pMCMC** |
| --- | --- | --- | --- | --- | --- |
|  | Intercept | -0.198 | -1.275 | 0.818 | 0.689 |
|  | Gut-passed, feces removed | 0.181 | 0.026 | 0.329 | 0.021* |
| Germination | Weight z | 0.134 | -0.177 | 0.458 | 0.414 |
| Probability | Age z | -0.005 | -0.096 | 0.088 | 0.914 |
|  | SexM | 0.326 | 0.169 | 0.493 | *<*0.001*** |
|  | ActivityNocturnal | 0.667 | -0.585 | 2.161 | 0.29 |
|  | Intercept | 2.322 | 2.091 | 2.569 | *<*0.001*** |
| Time-to- | Gut-passed, feces removed | -0.073 | -0.158 | 0.01 | 0.092+ |
| Germination | Weight z | -0.028 | -0.113 | 0.06 | 0.524 |
|  | Age z | -0.026 | -0.07 | 0.017 | 0.255 |
|  | SexM | -0.014 | -0.099 | 0.071 | 0.750 |
|  | ActivityNocturnal | -0.196 | -0.461 | 0.071 | 0.141 |

Table S9: Results for the lemur traits model in the captive experiments. *** indicates *p <* 0.001; ** indicates 0.001 *< p <* 0.01; * indicates 0.01 *< p <* 0.05, + indicates 0.05 *< p <* 0.1.

1. Revell, L. J. phytools: an R package for phylogenetic comparative biology (and other things). *Methods in Ecology and Evolution* **3**, 217–223 (2012).
2. Herrera, J. P. & Dávalos, L. M. Phylogeny and Divergence Times of Lemurs Inferred with Recent and Ancient Fossils in the Tree. *Systematic Biology* **65**, 772–791 (2016).
